# Fungal infection-related mortality versus total mortality as an outcome in trials of antifungal agents

**DOI:** 10.1186/1471-2288-6-40

**Published:** 2006-08-14

**Authors:** Anne K Due, Helle K Johansen, Peter C Gøtzsche

**Affiliations:** 1 Nordic Cochrane Centre, Rigshospitalet, Dept. 7112, Blegdamsvej 9, DK-2100 København Ø, Denmark

## Abstract

**Background:**

Disease specific mortality is often used as outcome rather than total mortality in clinical trials. This approach assumes that the classification of cause of death is unbiased. We explored whether use of fungal infection-related mortality as outcome rather than total mortality leads to bias in trials of antifungal agents in cancer patients.

**Methods:**

As an estimate of bias we used relative risk of death in those patients the authors considered had not died from fungal infection. Our sample consisted of 69 trials included in four systematic reviews of prophylactic or empirical antifungal treatment in patients with cancer and neutropenia we have published previously.

**Results:**

Thirty trials met the inclusion criteria. The trials comprised 6130 patients and 869 deaths, 220 (25%) of which were ascribed to fungal infection. The relative risk of death was 0.85 (95% CI 0.75–0.96) for total mortality, 0.57 (95% CI 0.44–0.74) for fungal mortality, and 0.95 (95% CI 0.82–1.09) for mortality among those who did not die from fungal infection.

**Conclusion:**

We could not support the hypothesis that use of disease specific mortality introduces bias in antifungal trials on cancer patients as our estimate of the relative risk for mortality in those who survived the fungal infection was not increased. We conclude that it seems to be reliable to use fungal mortality as the primary outcome in trials of antifungal agents. Data on total mortality should be reported as well, however, to guard against the possible introduction of harmful treatments.

## Background

Disease specific mortality is often used as outcome rather than total mortality in clinical trials. This approach assumes that the classification of cause of death is unbiased. However, a comparison of noncancer death rates in cancer patients with noncancer death rates in a matched population showed that use of cancer specific mortality may underestimate the mortality related to cancer, and that treatment-related deaths seemed to have been omitted from cancer mortality [1]. A review of cancer screening trials also identified inconsistencies between disease specific mortality and all-cause mortality [2], and it has been shown that biased misclassification of cause of death can give a spurious advantage to aggressive cancer treatments over less aggressive treatments [3].

For antifungal agents, a higher incidence of bacterial infections has been reported which might be a class effect related to the azole drugs [4], and itraconazole is associated with congestive heart failure [5]. As aggressive treatment may increase mortality due to treatment but decrease mortality due to the disease in question, bias in classification of cause of death may influence the results of the trials. According to the recommendations of the CONSORT Group all deaths should be reported regardless of cause [6].

In this study we explored whether use of fungal infection-related mortality as outcome rather than total mortality leads to bias in trials of antifungal agents in cancer patients. Most patients with invasive fungal infection are suffering from severe underlying conditions [7], and as fungal infections are difficult to diagnose, misclassification of cause of death may occur. Accordingly, in autopsy studies many patients were considered to have died from a fungal infection that was not suspected or confirmed antemortem [8].

## Methods

We used a sample consisting of trials included in 4 systematic reviews concerning prophylactic or empirical treatment in patients with cancer and neutropenia at risk for fungal infections, which we have published previously [4,9-11]. According to our protocol, trials had to be randomised, published as full papers, include mainly cancer patients and report on at least one fungal death and one death from another cause to be included in the study. Details on overall mortality and disease specific mortality were extracted by two persons independently and disagreements were resolved by discussion. Data were analysed as relative risks [12] and 95% confidence intervals (CI) are presented. A fixed effect model was used since there was very little heterogeneity between the studies.

**Figure 1:**
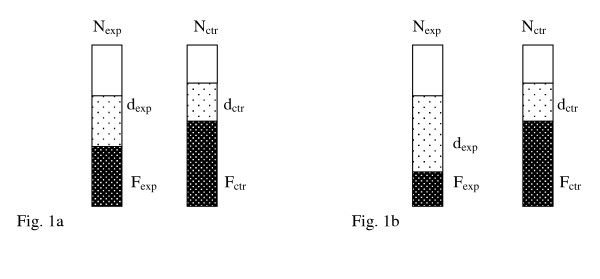
**Relative risk of death from causes other than fungal infection in those who survived the fungal infection**. Provided the two groups are still comparable after subtraction of fungal deaths from those randomised, the proportion of those who died from another cause than fungal infection would be expected to be the same in the two groups (fig. 1a). If, however, there was a bias in classification of cause of death in favour of the treated group, fewer deaths would be ascribed to fungal infection and more deaths to other causes in this group. This would result in a larger relative risk of death for those who did not die from fungal infection (fig. 1b). This risk is an estimate of bias overall: RR = (d_exp_/(N_exp_-F_exp_))/(d_ctr_/(N_ctr_-F_ctr_)), where exp: experimental group; ctr: control group; N_exp_, N_ctr_: numbers of randomised patients; F_exp_, F_ctr_: numbers of deaths from fungal infection; d_exp_, d_ctr_: numbers of deaths from causes other than fungal infection.

As an estimate of bias we used relative risk of death in those patients the authors considered had not died from fungal infection. Provided the two groups are still comparable after subtraction of fungal deaths from those randomised, the proportion of those who died from another cause than fungal infection would be expected to be the same in the two groups (fig. 1a). If, however, there was a bias in classification of cause of death in favour of the treated group, fewer deaths would be ascribed to fungal infection and more deaths to other causes in this group. This would result in a larger relative risk of death for those who did not die from fungal infection (fig. 1b).

## Results

Our sample consisted of 69 trials [13-81] 30 of which met the inclusion criteria [13-42]. Thirty-one trials were excluded as they did not report the necessary mortality data, 4 were published as abstracts and in 4 trials the patients did not have cancer. The interventions were no treatment, placebo, amphotericin B, fluconazole, miconazole, itraconazole and nystatin. In all trials that compared two drugs, it was easy to decide which was the experimental one and which was the control drug.

The trials comprised 6130 patients and 869 deaths, 220 (25%) of which were ascribed to fungal infection. The relative risk of death was 0.85 (95% CI 0.75–0.96) for total mortality, 0.57 (95% CI 0.44–0.74) for fungal mortality, and 0.95 (95% CI 0.82–1.09) for mortality among those who did not die from fungal infection (see additional files 1, 2, 3 for graphs and data extracted from the studies).

## Discussion

We could not support the hypothesis that use of disease specific mortality introduces bias in antifungal trials on cancer patients as our estimate of the relative risk for mortality in those who survived the fungal infection was not increased. We had expected some increase, even in the absence of any misclassification bias, since, in case of positive treatment effects, more severely ill patients would survive in the experimental group which would be expected to increase their risk of death, compared with surviving patients in the control group. It should be noted, however, that the confidence interval for our risk estimate, 0.82–1.09, is compatible with the possible existence of minor bias.

Subgroup analyses should generally be discouraged when a null hypothesis of no difference (no bias in our case) could not be rejected. However, we did an exploratory analysis where we included only those trials that were not blinded since the risk of bias is largest in these trials. The total number of deaths among those who survived the fungal infection was 255, as compared with 649 for the corresponding analysis for all the trials. The relative mortality risk among those who did not die from fungal infection was 0.90 (95% CI 0.72–1.14), which is very similar to our estimate of 0.95 (95% CI 0.82–1.09) for all the trials.

## Conclusion

We conclude that it seems to be reliable to use fungal mortality as the primary outcome in trials of antifungal agents. Data on total mortality should be reported as well, however, to guard against the possible introduction of harmful treatments as we cannot know whether our findings will apply to future antifungal agents.

## Competing interests

The author(s) declare that they have no competing interests.

## Authors' contributions

PCG conceived the study; AKD wrote the draft protocol and manuscript; all authors contributed to data extraction, writing the protocol and the manuscript, and approved the final manuscript.

## Pre-publication history

The pre-publication history for this paper can be accessed here:



## Supplementary Material

Additional file 1 **Total mortality **Graph and details on the computation of RR for total mortality Click here for file

Additional file 2 **Fungal mortality **Graph and details on the computation of RR for mortality from fungal infection Click here for file

Additional file 3 **Other mortality in survivors of fungal infection **Graph and details on the computation of RR for mortality in patients classified as not dying from fungal infection Click here for file
