# Supplementary figures and images for: Fungal infection-related mortality versus total mortality as an outcome in trials of antifungal agents
Source: BMC Med Res Methodol. 2006 Aug 14;6:40. doi: 10.1186/1471-2288-6-40 (PMC1559710; doi:10.1186/1471-2288-6-40)

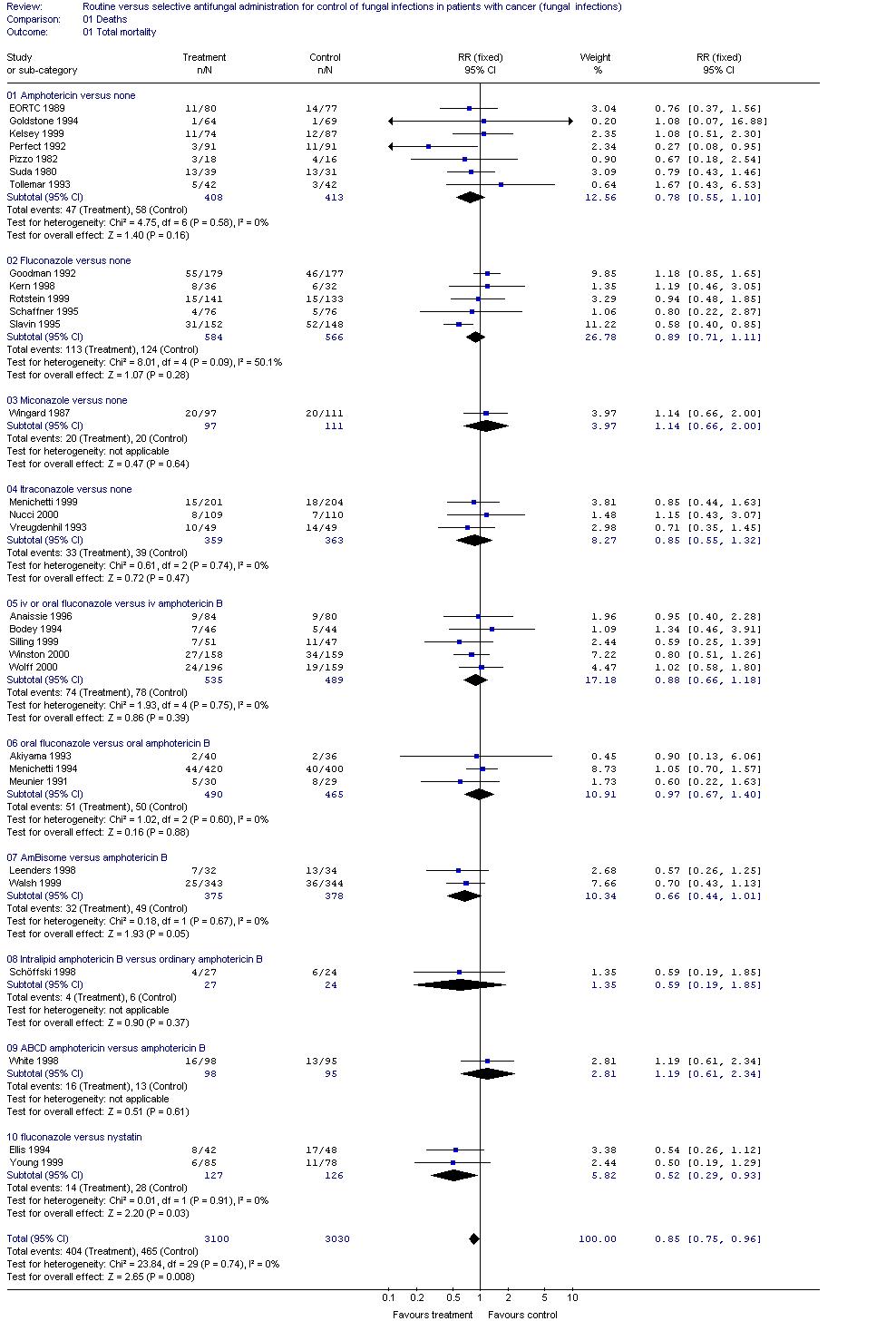

Supplement: Additional file 1 — Total mortality Graph and details on the computation of RR for total mortality [file 1471-2288-6-40-S1.jpeg]

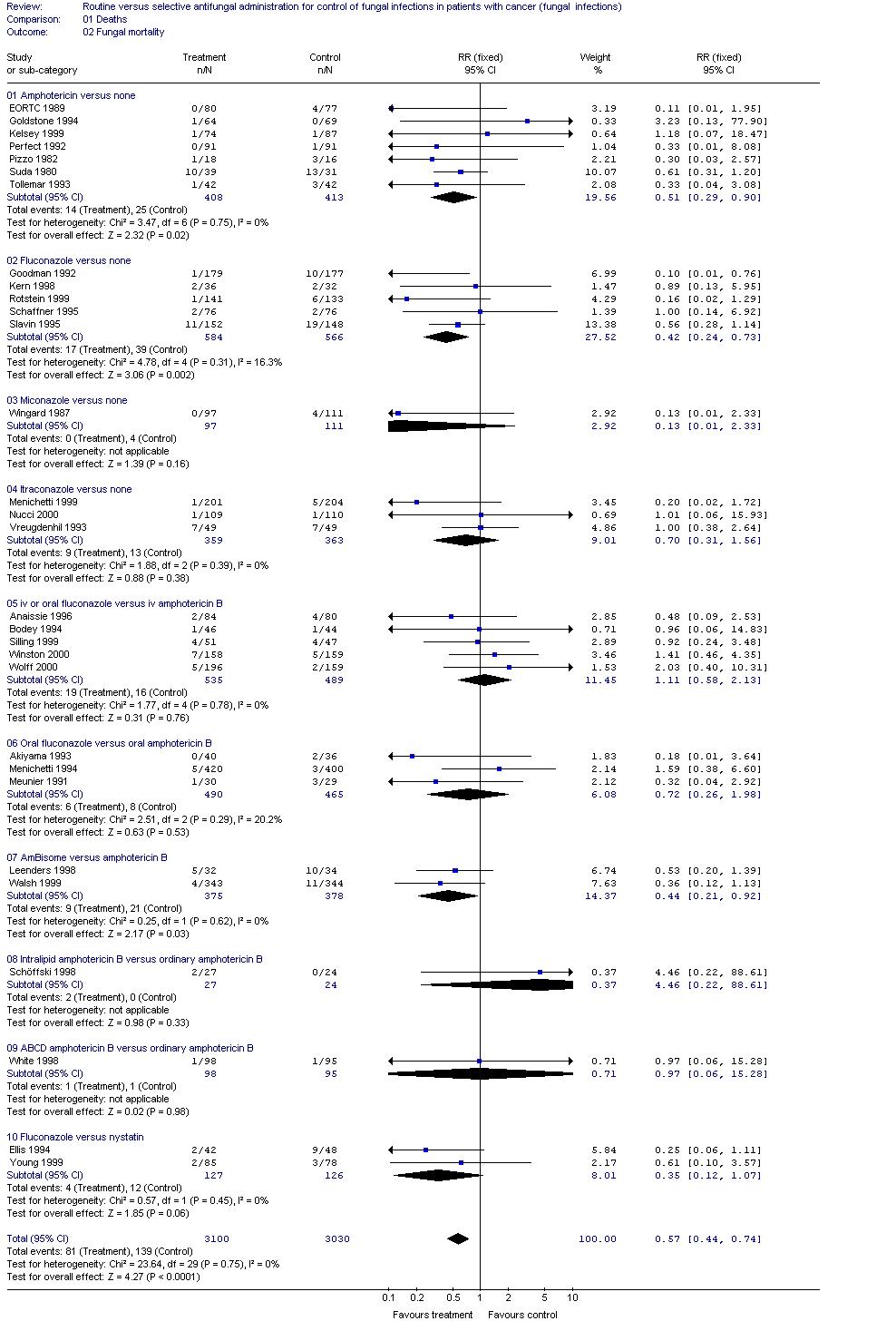

Supplement: Additional file 2 — Fungal mortality Graph and details on the computation of RR for mortality from fungal infection [file 1471-2288-6-40-S2.jpeg]

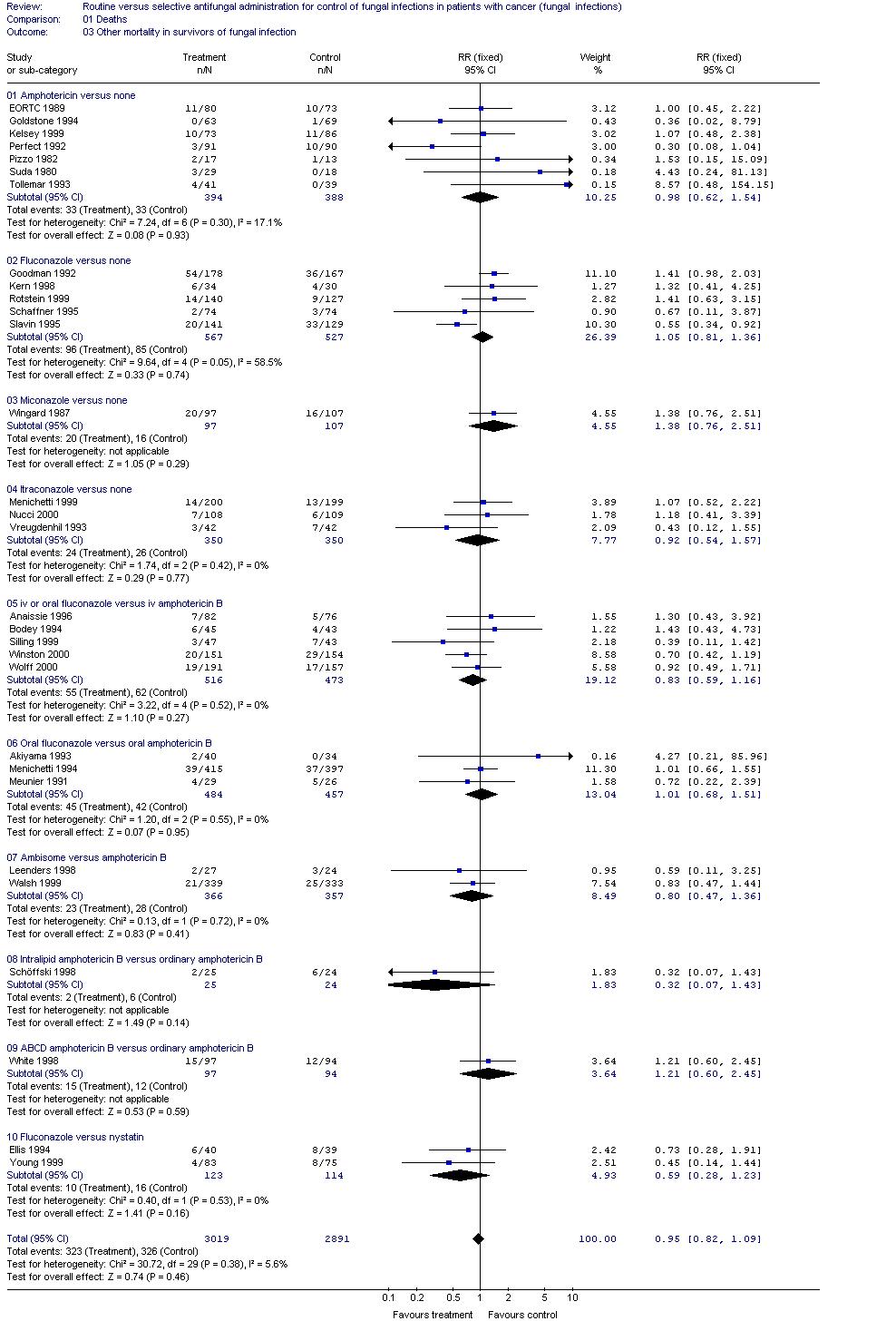

Supplement: Additional file 3 — Other mortality in survivors of fungal infection Graph and details on the computation of RR for mortality in patients classified as not dying from fungal infection [file 1471-2288-6-40-S3.jpeg]
